# Situated imagination

**DOI:** 10.1007/s11097-020-09701-2

**Published:** 2020-09-03

**Authors:** Ludger van Dijk, Erik Rietveld

**Affiliations:** 1 Centre for Philosophical Psychology, Department of Philosophy, University of Antwerp, Antwerp, Belgium; 2 Amsterdam University Medical Center, Department of Psychiatry, University of Amsterdam, Amsterdam, The Netherlands; 3 Department of Philosophy/ILLC, University of Amsterdam, Amsterdam, The Netherlands; 4 Department of Philosophy, University of Twente, Enschede, The Netherlands

**Keywords:** Affordances, Ecological psychology, Enaction, Embodied cognition, Imagination, Skilled intentionality framework, “Higher” cognition

## Abstract

Imagination is often considered the pinnacle of representational cognition. Looking at the concrete details of imagining in context, this paper aims to contribute to the emerging literature that is challenging this representational view by offering a relational and radically situated alternative. On the basis of observing architects in the process of making an architectural art installation, we show how to consider imagination not as de-contextualized achievement by an individual but as an opening up to larger-scale “affordances,” i.e. the unfolding possibilities for action. We show how the architects coordinate the enactment of multiple affordances across different timescales, from small-scale affordances of picking up a mobile phone to the large-scale affordance of making the installation that takes months to unfold. These affordances get co-determined as they are jointly enacted. It is within this determining process that imagination too finds its place. On our view it is the indeterminacy of multiple affordances unfolding in action simultaneously that can be experienced as imaginative. The indeterminate character of this coordinative process allows activities to widen and open up, letting new possibilities for action enter into them.

In order to see more clearly, here as in countless similar cases, we must focus on the details of what goes on; must look at them *from close to*. (Wittgenstein 1953 §51)

## Introduction

Radically situated approaches to cognition have slowly been gaining momentum. After modern enactive and ecological theories made headway in areas such as perception and “basic” activities (Chemero 2009; Hutto and Myin 2013) and developed the notion of “affordances” to extend to sociomaterial practices (Gibson 1979; Costall 1995; Rietveld and Kiverstein 2014; Heft 2007), they have recently started to approach imagination in non-representational terms (Gallagher 2017; Hutto and Myin 2017; Ingold 2013; Rucińska 2016; Van Rooij et al. 2002). The basic scheme to account for imagination has two ingredients, neither of which is sufficient by itself. First, there is the history of prior situated activity that sets up the individual with the appropriate sensitivities (Gibson 1979, p. 255 ff.; Hutto and Myin 2017, p. 195). Second, this history is ongoing and continued in the current concrete situation that the sensitive organism is now engaging (Gallagher 2017, p. 194). This concrete situation is not only constraining what can be done by the individual, but is also enabling activity by providing multiple opportunities for engagement, or “affordances” (Gibson 1979, p. 127; Rietveld and Kiverstein 2014; Gallagher 2017). The individual’s situation constitutively includes a wide context of engaging with opportunities for action offered by other people, materials, tools, written text or conversation.

What makes such a proposal “radical” is the reversal at the heart of it. Imagination is situated and temporally extensive. Imagination is not considered a determined state inside the individual, which resulted from a history of interactions to now cause a new action. Rather, it is integral to a temporally extensive process: a coordinative process that includes a history of activity, is currently engaging a wide practical context, and anticipating its way into the future (Van Dijk and Rietveld 2018). On this view the bodily sensitivities of the organism, such as a sensitized visual system (Gibson 1979, p. 255), are necessary but not sufficient for imagining. Imagination takes shape in a *temporally* constituted process (Kirchhoff 2015; Gallagher 2017, p. 8; Van Dijk 2020). In such a process the relations forming are not logically preceded by the existence of their relata – rather, relation and relata mutually take shape together over time. Consequently, what is being imagined is constitutively tied to the history of activity *and* the possibilities for future activity available. In what follows we shall not argue for this reversal (see e.g. Hutto and Myin 2013; Ingold 2011). Rather we focus on adding to the development of the radically situated approach that the reversal implies.

In our view there are two problems with the radically situated proposal. In its current form it is both too philosophical and not philosophical enough. First, it is too philosophical in that it glosses over many important details. Indeed, it is widely granted that the material environment shapes our imagination (Malafouris 2013; see Hutto and Myin 2017, p. 195; Gallagher 2017, p. 192 ff.). Still, imagining is seemingly afforded all the time, so we need to account for when specific situations *invite* individuals to imagine. Relatedly, there seem to be situations in which imagination is characterized exactly as *not* being responsive to the materials in our immediate environment. For instance when stopping whatever one is doing, stepping back, and reflecting in order to find continuation. How does a situated account to imagination deal with such cases? The second problem is that the basic account sketched above isn’t philosophical enough. As a philosophy of the particular, radically situated approaches should not contend in a general characterization of imagination. The details of our engagement matter in each particular case (Dutilh Novaes 2013; Mol 2002; Suchman 2007).

Our solution to these problems is to supply a philosophical analysis of an actual, real-life case of imagination. Our starting point is the processual notion of affordances that emerged from pragmatist developments in ecological psychology (for details see e.g. Shotter 1983; Costall 1995; Heft 1989, 2007; Reed 1996; Rietveld and Kiverstein 2014).^1^ We developed this processual view of affordances further in earlier work (Van Dijk and Rietveld 2018; see Section 2 below). Using a processual approach, in which affordances are determined in action over time, we aim to foreground the richness of the situations in which imagination may occur. Minimally, this will put some meat on the bones of situated approaches to imagination. Our philosophical fieldwork in the practices of visual art and architecture that we will present below involved prolonged observations that gained us an eye for the temporally extensive processes in which imagination occurs. Through these observations we aim to foreground the *indeterminacy* of the situations that organisms engage in real-life. Thus we moreover aim to develop the above conceptual reversal with a philosophical analysis of the details that matter for imagining in concrete situations.

In the following we will offer a philosophical ethnography of imagination in architectural practice. After explaining the theoretical and methodological background to our approach in Section 2, we will present our philosophical observations of architects imagining (during their work of designing an architectural art installation). We do this initially in terms of the larger-scale process of making that unfolds and gets more determinate over time (Section 3). Then, in Section 4, we will continue the observations but focus on the role of the individual participating in this process. Section 5 will present the philosophical view of imagination that emerges from our fieldwork. We will explicate how imagination is an integral aspect of skillfully coordinating to multiple affordances across timescales. It is the indeterminacy of that process, a process in which a manifold of affordances contribute to each other’s enactment, that a participating individual can experience as imaginative.

## Foregrounding the process

We will use “imagination” to refer to a family of phenomena that cognitive science aims to account for, including, but not limited to, imagining what a not (yet) existing work of architecture might look like, deliberately thinking about the environment as being different from how it actually is right now, but also for example the experience of mind-wandering. We aim to show how such examples can be fruitfully re-approached by understanding them as involving continuous coordinating with extensive, large-scale processes that the organism is simultaneously participating in. Tentatively, we will suggest that it is the indetermination of affordances unfolding in action that is experienced as imaginative by the organism involved. This will be more pronounced for affordances unfolding over a large timescale. We will draw out several examples in which the particulars of the situation invite participants to draw attention to such large-scale affordances, in terms of imagination. Our ethnographical examples will work towards a more detailed account of imagination in Section 5. The current section will explain our theoretical background as well as the methods we used, and end with introducing the architectural practices we observed.

### Temporalizing affordances

Coordination consists in ongoing, world-involving activity that unfolds across multiple timescales. In this process one participates in the enactment of multiple affordances. Affordances on our view are sociomaterial, forming as materials and people meet. For instance, to an architect coordinating in architectural practices in which sponges are used in scale models of public parks to represent trees, a sponge can afford multiple actions simultaneously: to be dyed green, to cut it to shape, and to stick a tooth pick in. In so doing, the affordance of making a model tree is enacted. Making the model tree is a larger-scale activity that consists in coordinating the smaller-scale activities of cutting, dying and sticking a tooth pick in. The affordance of making the tree thus unfolds over time and holds together in practical engagement. An affordance can thus be thought of as constituted across multiple timescales of activity (Van Dijk and Rietveld 2018).

**Fig. 1:**
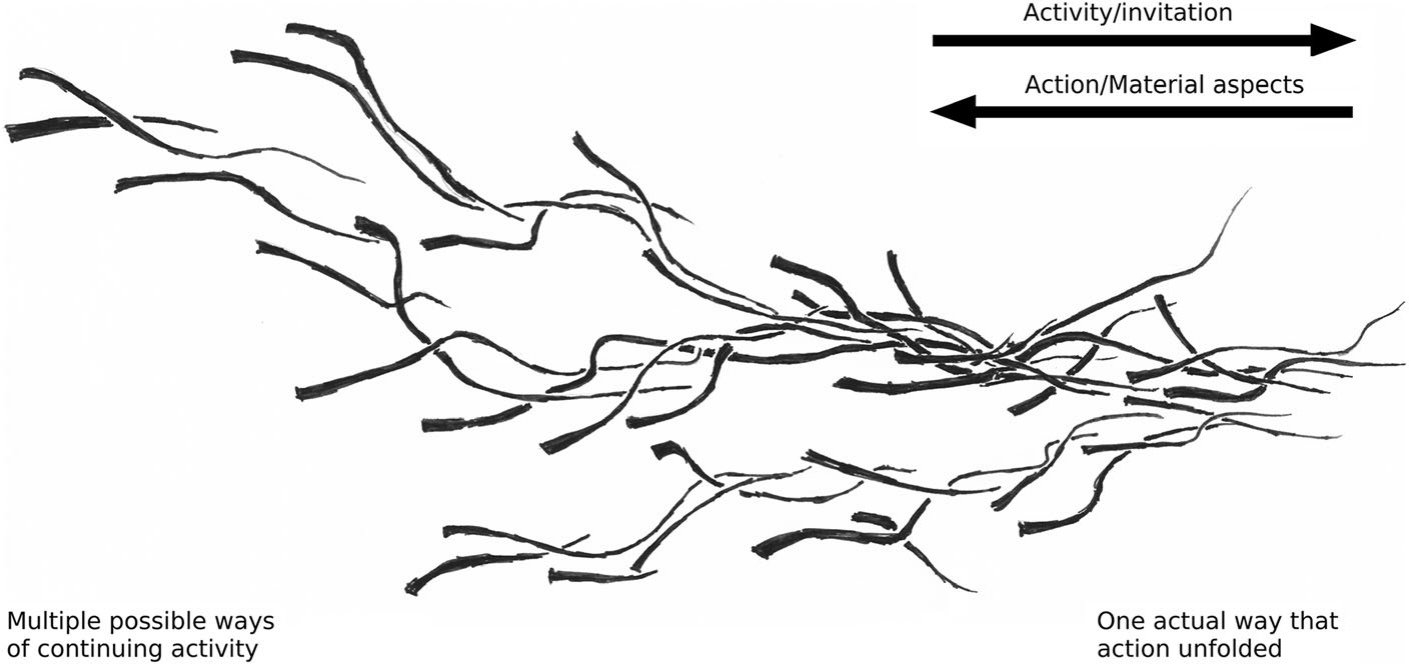
(Taken from Van Dijk and Rietveld 2018). Affordances unfolding as a multi-scaled process. Each line represents an affordance unfolding in activity. The use of lines aims to emphasize that, in process, affordances are actively determined over time and have a direction of unfolding (Heft 1989; James 1912), The single lines intertwine to form strands (here at least three strands can be identified). These strands will be identified in Section 4 as “(unfolding) situations,” with their own global direction. Strands in turn intertwine to form a larger-scale unfolding process – consisting of the figure as a whole (which also has a direction). At all these scales, the process is constrained by its own history, represented by their decreasing width. From left to right, the multitude of possible ways in which materials can still be coordinated, and activity can continue, makes way for the one actual way in which material has been coordinated and the activity has unfolded. The lines that make up the figure can be read in two directions. Starting from the left-hand side, the line indicates an activity that is determining an inviting possibility, while starting from the right-hand side looking back on the line, it indicates a determined action and a constraining material environment (see Ingold 2011, 2013; Schatzki 2012; Shotter 1983 and Van Dijk and Rietveld 2018 for details)

Responding to small-scale affordances (such as that of sticking a toothpick in a sponge) allows the larger-scales (from the making of a model-tree to making a scale model of a public park) to keep going. Conversely, by simultaneously participating in the larger-scale (the affordance to make a scale model of a building), small-scale possibilities for action are *inviting* in terms of it (the sponge in context). Participating in large-scale affordances then consists in coordinating activity such that affordances across timescales are jointly enacted – a process of coordination that increases the *determination* of these affordances. For example, the dyed sponge invites sticking a tooth pick in, and engaging that affordance simultaneously determines the possibility of making the model tree. The process of participatory coordination, of actively determining multiple affordances concurrently across timescales is depicted schematically in Fig. 1.

The work presented in this present paper builds on Van Dijk and Rietveld (2018). In that work, the focus was on the determinacy of direction that unfolding affordances in process might get. It focused on how an affordance for making an architectural art installation unfolded over the course of many months. It was shown how to think of such temporally extensive processes as an affordance determining in action over time, similar in kind to affordances such as grasping a cup or dyeing a sponge, that unfold over a much smaller timescales. By participating in unfolding affordances, organisms can sense their direction of unfolding as ‘anticipation.’ Concurrently, the individual’s contributions to the unfolding keeps the affordance going so that it invites further activity. Affordances, that is, set up the conditions for their own continuation by inviting participation (Shotter 1983; Van Dijk and Rietveld 2018).

In this paper the focus is on the *in*determinacy that the process of affordances unfolding across various timescales may have. We shall propose that it is this indeterminacy that an individual participating in the multi-scaled process may sense and experience as imaginative. The remainder of this section will explain the method of observation we employed and introduce the practices of the architects that were observed. Section 3 will provide an overview that allows us to take note of the large-scale processes that individuals participate in, while we simultaneously keep an eye on the smaller-scale situation that unfolds to constitute that large-scale affordance over time. In Section 4 we will discuss some of the conditions in this process that might invite participants to draw attention to their imagination.

### Philosophical ethnography

**Fig. 2:**
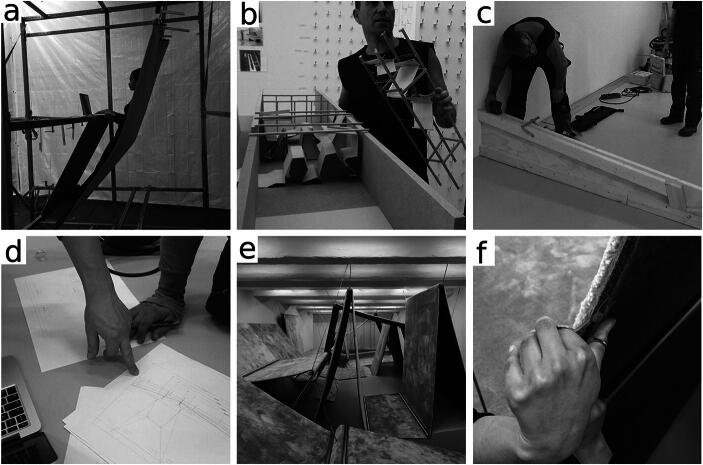
Examples showing some of the diversity in affordances within the practice of architects, giving a general impression of some of the activities unfolding when making an architectural art installation. **a** Exploring a carpet for supporting the back while standing and using it to support a laptop so that one can type. **b** Build models that invited comparison for different design features, notably the possibility to suspend carpet in the air. **c** Wooden beams that afforded making a tilted floor for optimal foot support and aligning it with the room where the installation was being built. **d** Measurements taken of models such as in Fig. 2b invited to make a schematized drawing on paper. **e** A photograph of the installation consisting of carpets suspended in the air by steel wires as it neared completion. By that time it invited only minor transformations to the architects, for instance (**f**), the edges of the carpet invited to be cut with a small pair of scissors (see Van Dijk and Rietveld 2018)

The fieldwork was carried out by one of the authors (LD) at a studio for experimental architecture called RAAAF and at several of their on-site locations over the course of over a year. The architects make site-specific work at the crossroads of visual art, architecture, and philosophy. At the time of the fieldwork the architects worked on making a new architectural art installation over a period of nine months. With this installation the architects investigated the possibilities of breaking the habit of sitting by creating landscapes of affordances that support living without chairs (the completed installation is depicted in Fig. 2e; for background see Rietveld 2016; Van Dijk and Rietveld 2018 and Section 2.3).

The aim of our fieldwork was to identify and make tangible concrete situations of abstract activity – of imagining, reflecting, anticipating and so on. Our starting point was that such activities are temporally extensive processes that could be observed only by being embedded in the practice of architects for an extended period of time (Ingold 2013; Mol 2002; Suchman 2007). ER was intimately acquainted with this practice as he works not only as a philosopher but is also one of the founders of the architects’ studio. This allowed for developing a method that we call “philosophical ethnography,” which consists in interlacing observations and philosophical work in a productive exchange. LD divided his time between the architects’ studio, or on site locations (a warehouse, shops, bars and exhibitions), and his office. In this way LD, as well as ER, would bring concrete observations to the discussions within their philosophical team and bring the philosophical works that were engaged to the observations of the architects at work, in hopes of transforming both practices.^2^

At the architects’ studio LD had unrestricted access to all situations. The architects consented to, and were aware that, LD was observing their activities. LD was observing the architects in their daily work. This included observing them making models, drawing pictures, planning appointments, sculpting clay, gluing paper, talking on the phone, and so on (see below). LD would join meetings and listen in on conversations. He made notes and took pictures or video on the fly, mostly by phone. Occasionally LD would participate actively in discussions. He would also be asked to join to participate or observe when the architects felt that this would be of interest.

Looking for events that would help foreground the temporal structuring of imagination, LD would analyze the notes and videos. Recorded situations were transcribed verbatim and LD and ER discussed the transcripts and videos in search of a description of the situations that best captured the engagement of affordances over time. The architects would be asked for clarification of situations if necessary. Over the course of months, in the back and forth of observations and philosophy, the most fruitful way of approaching situations in their larger practical context became clearer. So would our description of the recorded situations evolve to capture the temporal structuring of the phenomena that we sought. The final descriptions were presented to the architects for feedback. With their approval they have been included in the following sections.

### The architects at work

The architects were invited to create an art installation at the headquarters of the Mondriaan Fund, a national fund for the visual arts (“Art Fund” for short) in an old and monumental building. The artwork proposed by the architects should offer new ways of supported standing to the visitors and employees of the Art Fund and needs to afford solitary working, comfortable waiting (it replaces the former waiting area), as well as afford having a meeting with multiple people. With their work the architects aimed to question the “sitting society” and taken for granted sitting habits, by exploring the possibilities for a world without chairs. To the architects it was also important that the installation would be visually compelling and move earlier versions of their work on supported standing into new territory.

The form of life the architects grew up in, their skills and the expertise they gained from earlier collaboration on similar projects together with the commission from the Art Fund offer them a significant opportunity for action: the affordance of making a new art installation, which will be enacted over the course of many months. To give an impression of some of the activities unfolding, Fig. 2 shows some moments in the growth of the installation.

**Fig. 3:**
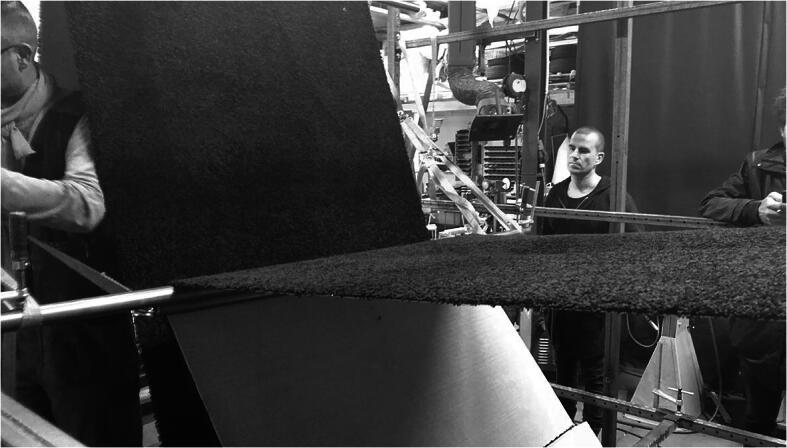
A metal frame in a warehouse, in which a strip of carpet is suspended using metal pipes and lashing straps. When this picture was taken a long pipe was getting replaced by a shortened pipe so here both can be seen holding up the carpet (see description of Part 2 for details). This position affords supported standing (see Fig. 2a)

The activities that we will focus on here unfold during the exploration of materials (following Fig. 2a). The architects involved are RR who is the one of the studio’s founding partners, junior architect DH and CS. At the time, the available space at the Art Fund had been explored several times by the architects. The architects had been experimenting with many different materials in a large rectangular metal frame (2.5 × 2.5 × 3 meters, made out of horizontal and vertical beams). Materials tested include rubber tubes, straps, wooden plates, and sheets of carpet (see in Figs. 2a and 3). The aim of the work in the frame was a real-life test and exploration of the affordances for supported standing, leaning and hanging offered by these materials as well as to discover the most interesting possibilities for creating new art installations.

## An overview of the process of making

Today, RR, CS, DH and ER join builder KS in the warehouse. KS has been building a “position” according to the instructions given to him by the architects (Fig. 3). The position, a single piece of carpet suspended by lashing straps, readily afforded supported standing and had already been tested before in the warehouse (Fig. 2a); it was found to be well suited for the installation at the Art Fund. Based on prior experiments and calculations the architects have revised several details of the design, including the number of lashing straps that would be admissible to make it work. As we shall see, this latter revision had not been shared with builder KS prior to their arrival. The architects wanted to make the position anew however, because they wanted (i) to check if this position would offer a comfortable way of stepping in and out of, and (ii) to see what the effects would be of using cables to fixate the position in the room at the Art Fund. In the episode below we are going to follow the position’s development at the warehouse over about 30 min.

KS has been building the position before the architects arrived by suspending a long piece of the carpet in the air within a large metal frame using bar clamps, lashing straps and metal pipes (see Fig. 3). The metal frame was made at an earlier stage to be able create, explore and test out positions in it.

Let us first give an overview of the episode. We have numbered the eight parts (Part 1–8), so that we can refer to them below. In the overview we have italicized some of the lines that exemplify aspects of the process that we will address more fully in the next sections.

(Part 1) The built position for supported standing (Figure 3) invites the architects, RR, DH and CS to explore it: bouncing with the material, stepping into it, standing in it, eyes open and sometimes closed. Feeling how their feet and their backs are supported by the carpet. They try to work on a laptop in the position, and circle around the metal frame. The frame invites ER to lean on it. The tension and the slope of the suspended carpet afford to be fine-tuned until they are *just right*. Measurements are taken of the result for later use.

(Part 2) KS shows RR the horizontally placed circular metal pipe that is used to fold the carpet and holds the structure up. It extends *too far* on both sides, which actually makes it easy to get into the position because one can hold onto it. The installation to be realized will however not afford using such a long pipe for aesthetic reasons. Uncomfortable with the pipe, *RR wants to see clearly* how the position will be in reality: “Actually we should make it right now like that. So could we cut off the pipe? ‘Cause I don’t want to be surprised later on. Can we, can we do it right now?” A new pipe is measured and sawed to the right size by KS, and a metal ring is welded to it for attaching the straps to.

(Part 3) The new pipe with the nylon lashing straps afford attaching to the frame by KS and RR. The straps now make less of an angle. RR notices this and says: “And then *you have to imagine* this whole cable is going through the room, hé.” The pipe he holds in is hand invites to be held parallel to the straps to indicate the direction of a future steel cable that will replace the strap in the end. This invites ER to suggest such a cable would be useful for holding onto as one gets into the position, which invites DH, KS and RR to step into the position, which seems to go alright.

(Part 4) Two intermingling discussions unfold.^3^ The position invites DH to briefly discuss attaching the straps higher on the frame with KS – this option would skew the whole position and would require additional cables running into the floor. The position with the shorter pipe invites RR and CS to step into it and then continuing to consider other aspects of the position (which they like) as well as discussing the problem of the cable with ER. RR remarks: “*the cable is a nightmare* … it will kill you if you walk through the room.”

We will unpack what happened in these four parts in the remainder of this section. The subsequent parts, Part 5–8, we will discuss in more detail in Section 4. To give a sense of the whole episode, we however recount these parts here:

(Part 5) To solve the dangers of the cable running horizontally, several metal cables lying around in the warehouse invite comparison among each other and relative to previous encounters with cables. Discussing the whole design they had worked out also affords the architects to consider rotating the position within the whole design, or adding a vertical pillar. These options however *afford little more than a quick rejection*: RR declines them resolutely.

(Part 6) RR and colleagues remain content with the position and the tactility of the carpet. The carpet in front of him affords RR to feel it, even putting his face on it: “but this is yeah, *this is great*. You can also do a little nap like this” he says jokingly. Unclear how the position affords a solution to the problem of the cable, the position invites checking the position’s size to see if the width corresponds to its design specifications, and RR calls on DH for the measurements. They turn out to be fine: “*No, the problem is the cables*” ER is invited to say, RR agrees: “The real problem is the cables.”

(Part 7) DH has been invited to join the discussion and this invites him to discuss *a previously uninviting opportunity* provided by the frame, to attach the straps higher: “[KS, KS, should we not try it nonetheless?]”^4^ But KS had already tried this before the architects arrived and knows that this will skew the position unless extra cables are attached below. KS shows this to RR by attaching the straps higher. The position (with RR and CS in it) gets skewed and this affords KS to note that this can only be solved by an extra cable, which he attaches to the pipe, along the direction of the upper strap. “For this position it’s no problem” RR says while KS tensions the straps and the position re-centers. RR: “[Yes, then we’ve solved the problem, right?] I think then we solved the problem.”

(Part 8) Now however the tension on the carpet *does not get high enough*. The strap below would need to be pulled perpendicular to the strap above, DH notes, which would make it harder to step into the position and, moreover, people might trip over the cable. KS and DH together re-position the lower strap nonetheless. RR takes out his phone, looking for their most recent image of the cardboard model of the entire installation and finds in that image the place where the lower cable will be in the Art Fund space, and says to CS: “[Well,] *you should imagine that it’s here*. … It’s exactly at this spot then that it ends up”. CS further specifies the point in the image. “But then it’s no problem, hé. I think, if it if it ends up there,” RR says while zooming and pointing on his phone, “then ... it will be possible.” Thinking of the visitors at the Art Fund, he says: You don’t come here, you don’t walk here. So for this position I think we can solve it.”

We view this full episode, this situation, as a constitutive part of a larger-scale process of making an installation. This larger-scale process crops up at every turn in small-scale activity: in making measurements (Part 1), in noting the pipe is too long (Part 2) or that the strap is too horizontal (Part 3–5) or in taking out a cell phone and comparing images (Part 8). Such small-scale activities *invite* in terms of the larger-scale process. Concurrently, the enactment of these small-scale activities continue the larger-scale process in the current situation and thus contributes to its unfolding.

As the situation unfolds, the number of possibilities open to continue the larger-scale process of making the installation (of which the described situation is a smaller part) decrease. Overviewing the episode a multitude of possible ways the position might become part of in the architectural installation is slowly constrained until one particular way it affords to do so invites further continuation of the process. Allied with this increasing determining of the situation, what the position solicits at the start of the episode (Part 1), feeling, bouncing, measuring, is completely different from the ending of this example (Part 7 and Part 8) – adding a strap, comparing it to the image on the phone to determine that the use of cables is afforded in the context of the entire installation at its future location.

### Zooming in: Divergence and convergence of paths

Apart from a globally increasing determination of the process of making in this 30 min episode, looking closer we also witnessed a different phenomenon: some smaller and larger-scale movements go in diverging directions and afterwards they converge again. For example, after the problem of the horizontal cable is seen (Part 3), small-scale affordances unfold in several directions: the architects start measuring the carpet, feeling the materials and comparing the thickness and color of possible cables (Part 4-Part 6), before solving the problem by adding an extra cable below (Part 7). When a problem arises none of these previously relevant smaller-scale affordances that invited RR or his team move the process in such a way as to add to the enactment of the larger-scale affordance – in this case the affordance of making the installation as a whole in the way that it is visualized in the image on RR’s phone (Part 8).

As the situation by now does not invite the responsive individuals a quick re-directing. If this continues the individual taken up in the process would risk losing their attunement to the direction of the process of making. Participating in this large-scale process allows RR to sense how the situation is diverting from it. To see this, let us zoom in on the example outlined above. After replacing the horizontal pipe (Part 2) and attaching the straps (Part 3):

KS is putting tension on the carpet. Concurrently the bar clamps afford removing them from the old pipe by RR. RR steps back from the metal frame to overview it for several seconds. The strap, that now runs more horizontally, constrains the direction in which the process of making can develop. It invites using a cable that runs through the whole installation. Responsive to this, and to KS’s and ER’s presence, the situation invites RR to point out this constraint on the development of the installation to everyone, by talking, pointing and gesturing: “And then you have to imagine this whole cable is going through the room, hé” he says. Saying this affords him to explain the constraint to ER, holding the pipe parallel to the straps to indicate the direction of the required steel cable while doing so.

RR sees the constraint that the strap poses on the overall development of the installation because he is responsive simultaneously to both the affordance for continuation that the position in the metal frame offers as well as to the entire process of making the art installation at the Art Fund. This responsiveness allows him to sense yet another affordance which becomes relevant in the situation: that of pointing out or articulating this discrepancy. This latter affordance, in this case, is enabled by the (responsiveness to) diverging scales of coordinating: “And then you have to imagine this whole cable is going through the room” RR says.

RR’s talking coordinates everyone with the affordance of the strap and the position in relation to the larger process of making: i.e. the constraint this solution will pose on the design and use of the installation. The others open up to the relevance of this aspect of the entire process too. They continue a path from the Art Fund space they are familiar with and is going to house the installation, the models and images of the installation that they made and so on, up to the current unfolding situation. That is, RR’s utterance and gesturing, in this situation, takes the horizontal strap up into a larger activity – his activity re-situates the strap by making use of other affordances the situation offers.

By coordinating activity such that the affordances of smaller and larger timescales are jointly enacted in the situation, the number of possibilities open to continue the making of the installation can decrease. As the example shows however, such coordination is far from easy. It requires skill and responsivity to the direction of the process, and a sensitivity to the change in determinacy that their activities may produce. In as much as skilled individuals contribute to the process, the process can keep inviting other activities from its participants to continue the process in the right direction (replacing a metal pipe, reconnecting the straps). The individuals engaged need to be responsive to any change in determinacy that might come with “divergence” in direction of unfolding scales of activity that a particular situation might engender. An example of such divergence was the activity of attaching a nylon strap that ran too horizontally and posed unwanted constrains on the development of the process of making. Let us change our focus to the other part of our situated approach to imagining, and look into the responsiveness required of an imagining individual situated in the process.

## Opening up to indeterminate possibilities

So far we have sketched the (temporal) context in which imagining occurs. In our case this is the large-scale affordance of making an art installation, as it is on its way to being determined in the activities that it invites from its participants. Turning these participants’ attention to the indeterminate large-scale unfolding is an activity that was itself invited by, and in turn contributes to, furthering that process. The imaginative activity in our example was not a de-contextualized or isolated achievement by an individual but was rather an opening up to a larger-scale affordance. It allowed the movement of the evolving situation to divert into several relevant smaller-scale activities: smaller-scale affordances of various kinds started inviting the architects and were explored for the direction these affordances could give to the overall process.

The question we want to answer in this section is why the situation invited one to imagine, that is, what invited attention to coordinative participation of this larger scale? To answer this question we discern three scales of the process. First the small-scale activities (a few seconds). Second, the “situational” scale of testing the position (about 30 min) and third, the large-scale of the process of making the art installation – taking about nine months. These different scales can run apart in several ways (Section 4.1 and 4.2) but are ultimately reciprocally constituted over time, as we shall see in Section 4.3.

### Maintaining coordination

In the process of making an art installation participants are invited in a concrete situation to continue in the direction of a large-scale process, while concurrently being responsive to any (lack of) converging of the direction of the smaller-scale activities enacted within it. In our example the situation in the warehouse, in which the architects are testing the different positions, constitutes the intermediate scale. This situation is reciprocally constituted, on the one hand, by the activities unfolding within it – the activities described above of checking one’s phone, talking, sawing a new pipe and so on. On the other hand, the situation reciprocally involves the large-scale making of the art installation, which is spread across many more situations (see Fig. 2).

In cases where the large-scale process is still going strong, but in which smaller-scale activities are diverging, the situation can invite quick resolution as new affordances become relevant. In such a case of noticing that something is “not right,” a skilled participant can experience “directed discontent” and immediately sense, often unreflectively, the relevant opportunities for improvement as inviting action (Wittgenstein 1967, p. 14; Rietveld 2008).^5^ In an architectural context, the skilled architect can thus sense the relevant opportunities for improvement based on a responsiveness to the affordances available in light of the larger unfolding process. In the process of optimizing the position that we have been focusing on, such quick, skillful redirecting can be identified throughout.

For example, in Part 1, the carpet is explored and the set-up fine-tuned by stepping into it, bouncing in it, feeling around, sensing how to tighten the lashing straps until remarking in a satisfied way “this is better, much better.” Similarly, in Part 7, KS adds a strap to re-center the position, but notices that one strap becomes too lose as the other tightens and the position does not center *and* sensing how the solution requires one of the straps to be attached further outwards. In such cases, in the context of a larger process, the situation smoothly invites the appropriate adjustments from the skilled craftsman or architect.

### A sense of losing coordination: A situation diverging

If however the activities have already diverted too strongly, or do so very quickly, the larger-scale unfolding *situation* itself might start to diverge from the direction of the process as a whole. Participants are then much more at risk of losing coordination with the large-scale process (they might even already have lost it), and thus, the situation may ultimately bring the continuation of the whole process in jeopardy (as it is reciprocally constituted by these situations). In our example, the process of testing the position (i.e. the whole unfolding situation) is diverging from the process of making the art installation. This is what happens when the use of the “deadly” horizontal strap turns out to hinder the continuation of the installation as the architects currently hope to make it. Cases in which the situation itself is not unfolding in the right direction offer clear examples of the sensitivity that participants have to the direction of such larger-scale unfoldings.

In such a situation the participating individual will feel “directed discomfort” at the lack of solution (Wittgenstein 1967, p. 14). The architects may not have a sense of how the available affordances can help them to return to act along with the entire process of making and may feel frustrated. In such cases, the process affords the individual little more than a “raw undifferentiated rejection” of the direction in which the situation might be moving (Rietveld 2008, p. 980). We witnessed such rejections, still afforded by the process of making, at several moments. For example, in Part 4 RR was being responsive to the possibility of a cable to run through the entire installation “the cable is a nightmare” RR expounded. Or again, when ER reflects that he should consider adding a (single) metal pillar to the installation (forking back to the installation in formation a few months ago) (Part 5). This suggestion affords RR to interject: “yeah, then yeah... As a design you are gone.” RR feels in other words, that doing so would change the direction of the forming installation beyond repair.

Note that these responses although still afforded by the continuing process, are not engaging or enabling adequate alternatives (Rietveld 2008). Sustained episodes of directed discomfort will therefore be frustrating to the individuals and may feel they are losing coordination with or grip on the larger-scale activity. Nonetheless, the utterances of discomfort are phenomena of, and contribute to, the movement of the installation – these expressions are as much afforded by the process as any other aspect of it. In particular, they constrain the process by pointing out how *not* to continue, and they scatter the unfolding situation, by pressing the individuals taken up by the process to *open up* even further to consider (previously excluded or neglected) affordances that could be relevant in light of continuing the larger-scale process of making.

In the observed situation, the prolonged discomfort had indeed opened the architects up to many affordances that were not yet relevant to them at the start of the episode. Engaging with these affordances allowed some re-directing of the installation in formation (for example, the exact width and color of the cables, that was decided on weeks later, was enabled in this unfolding), but for the most part, the directed discomfort continued for about ten minutes. Because the process was (frustratingly) unfolding in this way and because DH at this point was called upon for measurements of the carpet (Part 6), a situation ensued that invited DH to show what happens when the affordance of attaching one of the straps higher and adding an extra strap to the metal frame is enacted (Part 7–8). This would in the end lead to a solution of the problem of the dangerous cable.

KS and DH enacted the affordance of adding an extra strap to the metal frame together, together attaching the strap higher, and together disclosing what happens (Part 7): KS uses it to show how the whole position gets skewed and DH uses it to show how this then calls for the use of an extra strap. Adding an extra strap was not relevant in light of the process of making so far. In this dire situation however, the metal frame, the pipe, the carpet and the straps now solicit the addition of an extra strap, and in its enactment (Part 7-Part 8) a position developed that works (not only in this testing situation but also in light of past activities – the constraints encountered at the Art Fund, which have been transformed in models and images of the overall design). A path that directs the current situation to a fruitful continuation of the process of making is getting established.

To end our ethnography of imagination, let’s now focus on this active establishing of a path from past activities to the present situation and beyond. The point will be to show how imagination in process helps to amplify and determine the future direction of the unfolding process of making. Imagination turns out to be part and parcel of the situated activity of architects at work.

### Tying past actions back into the process

**Fig. 4:**
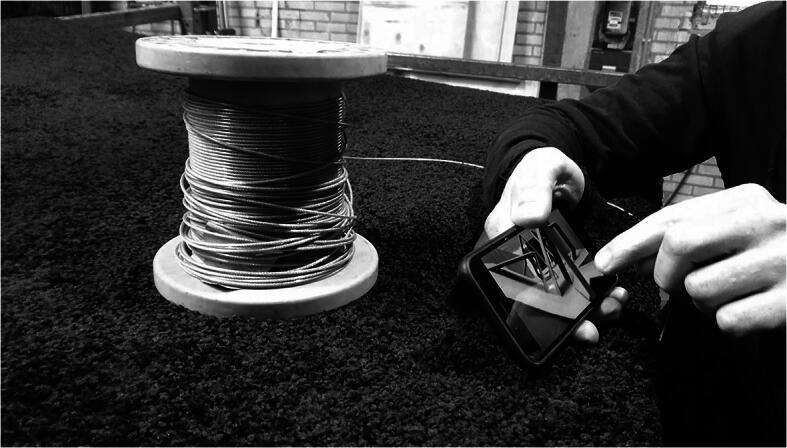
RR holding his phone while standing in the carpet with CS to his left (not shown). They are discussing the location of the cable in relation to a photograph of a cardboard model of their current favorite configuration of the installation as a whole (cf. Fig. 2b and e)

In Part 8 of the observations above, the new position the architects arrived at invites comparing it to the photos RR had of a cardboard model they have at the studio. RR’s phone affords such a comparison and he gets it out of his pocket (see Fig. 4):

“[Well,] you should imagine that it’s here.” The phone invites to be shown to CS. She responds, “hmhm,” inviting RR to go on: “there somewhere.” This invites CS to refine the use of the image: “hmmmm, it ends in between both” she says, pointing to a position on the screen. RR and CS situate the image further and further into the current situation: RR: “in between?” CS: “in between both ramps.” RR: “here”, “yeah” CS says. Thus the image invites comparing it to the position they are standing in: “But then it’s no problem, hé. I think, if it if it ends up there,” RR says while zooming and pointing on his phone, “then [...] it will be possible. [...] [Y]ou don’t come here, you don’t walk here. So for this position I think we can solve it.”

Note that the picture gets nested in the current situation, almost explicitly so. But that this in turn also situates the current activity in the entire process of making to which the photograph belongs. The old picture invites being used anew and can actively make the current situation continuous with the larger-scale unfolding.

By acting, responding to the phone’s invitation to compare images, in light of the process unfolding, the current situation is coordinated with the past. Or, to put it differently, the current situation continues a past activity by coordinating with the photograph from a past situation. This coordination requires work from the architects: it consists in actively re-situating the (old) image further and further into the larger-scale process: comparing the image to the position the architects are currently standing in, until a practical correspondence between it and the image is achieved in activity. The cardboard model of the installation shown on the phone, held in their hands and compared, forms the terms in which the current situation is viewed. The larger-scale is thus continued further in this activity.^6^

Actively comparing the image of the model, talking, gesturing, pointing, and thus translating the image into the current situation, the architects *achieve continuity* with the larger-scale process to which both the photograph and the position in which they are currently standing increasingly belong. By aligning their current activity to the image they achieve new and extended continuity from the activity of making the image to the current activity of attaching the straps to make the position work – the previous actions and the current activity form a new line to fruitfully continue further. In other words, both the current situation and the larger-scale process were amplified by situating the image into the current situation: doing so a new path is laid down from the making of the image to the current situation that adds momentum to the large-scale process of making and points to the direction of continuation.

Participation in the large-scale process and the possibility to align the current activity with it again invited the use of the word “*imagine*” (pointing to the image and saying: “you should imagine that it’s here,” Part 8), which establishes a path from the cardboard model and image to the current position. To reiterate, this required *more* involvement and *broader* coordination with the large-scale process rather than detachment from the unfolding activities. Coordinating with CS, RR succeeded in seeing the current position in light of the images, and cardboard models they had made of the installation in formation. By actively continuing their previous activities into their current activities, establishing a path along which their future activities could again continue. By doing so, their directed discomfort too was finally resolved and the installation could unfold further.

## Imagination in an unfolding process

Although our observations have been of a quite particular situation, many of the aspects we tried to highlight have more general applicability and refine recent radical approaches to imagination (i.e. Hutto and Myin 2017; Gallagher 2017). On our view, imagination is part and parcel of a temporal process in which inviting affordances across multiple timescales are constituted over time. As emphasized above, the process at any timescale has a direction and can increase determination. We’ve seen that a skilled individual is sensitive to a discrepancy in direction of the activities unfolding across different timescales. On our account such diverging of the process at different timescales creates indeterminacy in the overall process. An active individual constitutively tied up in this process, coordinating to affordances across several timescales simultaneously, “indetermines” with it. That is, if the process diverges or comes undone, then so do the individual in so much as it participates in it.

Imagination, we suggest, is an aspect of coordination with indeterminate processes that an individual participates in. Such coordination allows an individual to enact an affordance of one timescale in light of an affordance of another timescale. If both activities unfold in the same direction, concurrently getting determined together, then participating in the process, coordinating with it, will have a strong *anticipatory* character and may be experienced as such (Van Dijk and Rietveld 2018; see Gallagher 2017; Heft 1989). If, by contrast, the directions between scales differ, friction between unfolding affordances ensues, coordinating with, or participating in, this indeterminate process can be experienced as imaginative and as having a more “fictional” character (such as imagining a cable going through a room of an Art Fund by participating in the large-scale process of making an installation while holding a metal pipe and standing in a warehouse somewhere else).

### Coordinating with indeterminate processes

It is the indetermination of affordances unfolding in action that can be experienced as imaginative. When affordances are conceived as possibilities that get determined in actual activity in real life situations, any engagement with affordances can be more or less imaginative depending on the determination achieved already. Such indetermination is amplified by the multiplicity of affordances unfolding concurrently, reciprocally determining each other. When an inviting affordance is still early in the process of enactment (it is still largely indeterminate), coordination with this affordance, given the current situation, may be experienced as imaginative. For instance when the architects at the start of the project imagines what an installation might look like. Conversely, the further an activity has unfolded, the more determinacy and convergence across timescales, the less of an imaginative character engagement with an inviting affordance has. The invitation to “groom” the carpet (Fig. 2f) after the installation had been built is a nice example of this. What is invited, what needs to be done, is so clear that the architect can act without much imaginative experience.

More generally, the less determinate the enactment of an affordance is, because of the multitude of other relevant affordances unfolding concurrently as one acts, or because the affordance is enacted by coordinating activity across people and things (Costall 1995; Heft 2007), the more the participating in enacting those affordances can be experienced as imaginative in character. Conversely, the more determinate the environment and unambiguous acting on an invitation is, the less imagination is implied.

Recall the reversal we identified in the introduction: on radically situated approaches, cognitive aspects of activity were not considered a determinate state inside the individual that causes action (say some goal or “distal intention”), but they were considered *integral to a temporally extensive process of engagement*. Adhering to this view, our proposal suggests a way of approaching cognitive phenomena in their temporal context. To end this paper, let us consider two phenomena closely related to our case of imagination and how to re-approach them from a radically situated perspective. The first considers periods of absence, such as mind wandering, where it might seem that our processual approach is overly constraining as it ties it to a particular situation. The second case considers the converse: cases of deliberate planning, where one might think our view is too open-ended and does not yet constrain imagination enough.

#### Re-approaching situations detachment

Think of seemingly “detached” phenomena that are often described in terms of imagination. Such detachment may be attributed to RR when he (literally) took a step back from the metal frame and imaged the cable running through the room of the Art Fund (Section 3.1). But think also of moments in which we open up far enough that “our mind wanders” or even gets away from us (Ingold 2013, p. 69). For example, looking away from the sentences you’re writing in order to find continuation to the story, or other cases in which small-scale activity is finished and you are not immediately drawn into another, when an activity has no obvious continuation, or is too little of a challenge to keep inviting engagement (i.e. is boring to the particular individual). These moments of “absence” might easily make one think that imagination has no bearing on the current situation as that situation unfolds further.

Our ethnography has suggested that a narrow focus on these experienced moments of “absence” is misleading. We need to be mindful of the reciprocal constitution of the situation across multiple timescales that are easily ignored when describing such moments. This includes the timescales of the indeterminate activities that rely less critically on small-scale bodily movement but are still continuing in that moment and furnishes new possibilities for action. The processes of making an art installation or the story that you’re trying to write are examples of this: large-scale processes that reciprocally continue a history of skilled engagement. Our view suggests that moments of “detachment” are radically situated. With multiple timescales of continuous engagement being constitutive of those moments. We need not think of these moments as “representing” something absent or non-existing but can rather think of them as an experience of participating in an ongoing, still indeterminate, process. Rather than “detaching” from the process, imagination is more fruitfully thought of as *opening up* the participating individuals *further* to other affordances that the multi-scaled process of making also provides.

Although we have not foregrounded this aspect here, one particularly important activity is that of engaging linguistic practices. Situated in linguistic practices, imagination can be continued into the situation by talking, writing and so on. By letting oneself be invited to describe what one imagined, by partaking in conversation, indeterminate situations can invite activities that allow one to attend to the larger-scale process it grew out of. In such activities, imagination is continued into the situation by talking (James 1912; Dewey 1958). We saw this when RR articulated how to imagine the cable going through the room. Or think back to the case of discussing how to imagine the picture on the phone (see Section 4.3). As part of the situation, the possibility of talking or of using pictures can contribute to the phenomenological character of one’s imaginings. Being situated in linguistic practices or practices of drawing pictures need not imply that our active participation in these practices is internalized in a linguistic or pictorial form. It just means that our imagination is more readily continued into specific activities that do take such forms.

#### Goals in an affording process

One might grant us that by taking into account the multi-scaled, historical and ongoing, temporal context in which periods of absence get constituted, metaphors of “detachment” or “decoupling” lose their grip. That is, by temporalizing the situation, in effect changing or enriching what a “situation” consists in, we may have redressed the worry that our view is too constrained by current engagement. Yet there is also a converse worry one might have: that our view is not constrained enough. That a temporalized view would still need a, representational, supplement because else it would remain too open-ended to do justice to activities that are strongly constrained by explicable or articulable goals or plans.^7^

We’ve argued elsewhere that a long term project of making an art installation can be understood as enacting a large-scale affordance (Van Dijk and Rietveld 2018). Our aim there was not to deny a role of articulable goals in this process, but to foreground the temporality of a pragmatic, affording world in which goals may take shape (Suchman 2007; Gallagher and Ransom 2016, p. 344).

The architects obviously had goals for the long-term. They were aiming for a goal that they had agreed upon with the Art Fund, which was to make a follow up of the End of Sitting installation that would show the architects’ vision on the living environment of the future (Section 2.2). The architects created detailed plans that specified the design in working with the carpenter and other builders (Fig. 2d). They would equally specify goals to each other, stick to a schedule, match calendars, give verbal descriptions of the installation they imagined, and were often planning what to do next and when to do it. It is very well possible that for participants who grew up in linguistic practices, the experience in such a process of making, such as one’s imagining or anticipating, gains a reflective character. Their engagement may at times be experienced as “having a goal in mind,” or “knowing exactly what to do next” as participants are invited to contribute their skills to further determine the direction of the process.

Rather than simply assuming that a goal must therefore pre-exist the process to cause, and give continuity to, activity over time, a philosophy of the particular like ours would follow Lucy Suchman (2007) and approach the question of how to best understand the phenomenon of being guided by a goal by closely observing the place of long-term goals across actual situations. This amounts to looking at the temporal constitution of long-term goals by observing when and where the articulation of goals and plans is invited and what such activity in turn invites doing. Think back, for example, to the way the pictures of cardboard models (Fig. 4) that had emerged in explorative activity (Fig. 2b), in turn started to guide the discussion between the architects, by offering additional criteria for successfully solving the problem we saw in Section 4.4 (Fig. 4).

We intend to return to the details of linguistically structured processes and how they shape our imaginings on a different occasion. Our observations suggest however that finding continuity across articulated goals, written plans, images and models over time seems to be achieved in activity, while they also set up the conditions for the process to invite further activity. One’s sense of being guided by a goal, our view suggests, is neither preceding nor merely following this open-ended process. It consists in coordinating activity such that multiple affordances across timescales are jointly determined.

## Concluding remarks

On the basis of observing architects in the process of making an architectural art installation, we showed how to consider imagination as part and parcel of concurrently participating in the enactment of multiple affordances. We considered affordances to be in flux and constituted across several scales in action. While performing one activity, one is participating in the enactment of several relevant larger-scale affordances too. These larger-scale affordances will typically be less determinate. We suggested that we are able to experience our participation in these larger timescales, and coordinating with the affordances available in them, as imaginative.

Having offered a philosophical analysis of imagination in architectural practice, what we hope to have made plausible is that concrete situations, from transforming images of a cardboard model into wooden beams (Fig. 2d-c) and from suspending carpet in a metal frame to using words like “you should imagine that it’s here” (Part 8), can be understood in terms of coordinating with affordances that invite these activities (and, contributing to the larger scale process, with the affordances that thus start inviting next). We made an effort to develop a perspective in which neither the individual nor their materials were privileged at any point in time. We took a process-based approach in which (imagining) individuals and material aspects were the continuous outcomes of an open-ended and multi-scaled process.

Scrutinizing the particular and concrete is not a standard philosophical strategy. We however believe that if we wish to move beyond the traditional dichotomy between abstract thought and concrete activity fully, as the situated approach to cognition professes, we need to resist reiterating the dichotomy as a prioritizing of either philosophy or observation in our theorizing, let alone equating the former with abstraction and the latter with the particular. By presenting *prolonged observation of the abstract* in practice we showed by example the possibility of a *philosophy of the particular*. Through our methods we hope to have shown the delicate reciprocal intermingling of observation and philosophy over time, requiring and valuing both equally. Returning to where we started then, by the very act of writing this paper we argued how practices, from philosophy to ethnography and architecture, can intermingle to open up new possible ways of doing, and help us to start imagining imagination differently.

## Footnotes

1 It has notably been argued that Gibson’s ecological psychology was inspired by William James’ radical empiricism (Heft 2001; Van Dijk and Kiverstein 2020). With radical empiricism it shares a commitment that organisms experience the relation they actively establish in terms of what the environment affords them to do. This ongoing process exhibits a directional ‘tendency’ to which individual animals need to grow responsive (Heft 2003, p. 167). Affordances are thus said to “invite” or “solicit” us to act (Rietveld 2008; Withagen et al. 2012). A similar view on the experiential character of affordances can be found in work that explores ecological psychology’s relation to the phenomenology of Merleau-Ponty and Heidegger (e.g. Heft 1989; Käufer and Chemero 2015; Rietveld and Kiverstein 2014; Van Dijk and Withagen 2016) as well as to Gestalt psychology (e.g. Gibson 1971; Heft 2001; Kiverstein et al. 2019).

2 Indeed, the architects at RAAAF (Rietveld Architecture-Art-Affordances) themselves explore the concept of affordances in their work. In the long run, the philosophical projects thus also feeds back into the way the architects approach their subject-matter.

3 DH and KS discuss many details of the design as they go over the way KS has built the position and the specifications DH had send him, for example, comparing measurements of the position to the ones available on a laptop. This activity however unfolded out of sight of the observer (LD) and was detailed to him afterwards, it is not discussed here.

4 Sentences that were originally in Dutch are translated into English. These translated sentences appear in square brackets.

5 In this analysis of the architects at work, the focus is on a situation that wasn’t exactly running smoothly. We use these examples because the phenomena of directed discontent and directed discomfort make the sensitivity to multiple entangled affordances across timescales clearly visible. These are also the situations when participants may notice the tension they experience in the situation and articulate their worries in terms of their imaginings, which in turn serves to coordinate the diverging scales (see Section 5). We do not wish to claim that imagination happens only in case of a problem or “breakdown” (see Suchman 2007, p. 73). In situations of breakdown it is however comparatively easy to notice an activity, or a situation, diverging from the direction of the larger process, constituting the decreasing determinacy of that process.

6 Note that, on a process view, continuing the use of the image in a new way changes not only what the process can become but also, retrospectively, what the image was before. The image, considered as affording material, is itself on its way to being determined. It constrains, but is not finished. Continuing the history of use of the image thus changes what is the image is (Dewey 1958; Shotter 1983; Van Dijk and Rietveld 2018).

7 It has for instance recently been argued that our (2018) view requires the addition of “distal intentions” (Brancazio and Segundo-Ortin 2020). Without distal intentions, so the worry goes, we lack a story about what “pre-dates” activity and “links affordances across a timescale” (ibid., p. 4). That is, by not replacing the role usually served by representations the claim is that we leave a gap in our account that still suggests that “behavior [is] guided by something that sure looks … to involve an internal representation” (ibid, p. 7). Reading this criticism as a warning that, by not providing an account of articulable plans or goals, the subject could be co-opted by representational approaches, the article raises an important point. But the authors, at times, seem to do themselves a disservice by suggesting that a non-representational view of explicit planning will only work if it is grafted on the traditional representational model of cognition. Distal intentions mean to capture a particular experiential quality, in which situated activity has an explicable goal that is distant in time and/or space (Gallagher 2012). We agree that this experiential phenomenon needs to be acknowledged. However, distal intentions are traditionally also used to explain the phenomenon in terms of a mental stand-in that specifies the world it its absence (Pacherie 2008). We would worry that insisting on distal intentions to explain “why certain acts … happen instead of others,” i.e. to explain intentionality, may be circular (Brancazio and Segundo-Ortin 2020, p. 7; see Dewey 1958; Heft 2003; Shotter 1983; Hutto and Myin 2013; Van Orden et al. 2001; Gibson 1979, p. 253). It may moreover not solve the problem articulated by Brancazio & Segundo-Ortin (2020), as one might ask why *this* distal intension rather than another guided the process. We would urge to distinguish any explanatory role allotted to distal intentions from the experiential phenomena they may single out (ibid. p. 9). Thus, the reciprocal and ongoing processes that allow distal intentions to take shape might be properly foregrounded in the analysis of situated action. However Brancazio and Segundo-Ortin (2020) rightly stress the role of plans and goals in the human form of life. The formation of distal intentions is indeed a form of situated activity (ibid. p. 6). Just like any other activity they surely have their part in intertwining affordances (ibid. p. 7). On our view, as well as theirs, goals and plans are determined in the process of making, while they concurrently also determine that process (see ibid. p. 8). But for that very reason distal intentions do not “pre-date” the process in which they take shape, let alone do they need to be understood as having some causal “impetus” (ibid., p. 7; cf. p. 4; Heft 1989; Oyama 2000; Reed 1993; Suchman 2007; Van Dijk 2020; Withagen et al. 2012). In short, if affordances are constituted in activity over time, there is no need to add distal intentions that “link” affordances from outside the process. One can experience the directions of affordances unfolding, and the indeterminacies this brings, by actively contributing to their enactment. For language-using animals the experience of such participation may, in some situations, be that of being guided by articulable goals. We thank an anonymous reviewer for bringing this work to our attention.
